# Postnatal loss of the insulin receptor in osteoprogenitor cells does not impart a metabolic phenotype

**DOI:** 10.1038/s41598-020-65717-3

**Published:** 2020-06-01

**Authors:** John L. Fowlkes, R. Clay Bunn, Evangelia Kalaitzoglou, Phil Ray, Iuliana Popescu, Kathryn M. Thrailkill

**Affiliations:** 1 University of Kentucky, Barnstable Brown Diabetes Center, Lexington, KY, USA; 2 Department of Pediatrics, University of Kentucky College of Medicine, Lexington, KY 40536 USA

**Keywords:** Bone, Metabolism, Molecular biology, Physiology, Biomarkers, Endocrinology

## Abstract

The relationship between osteoblast-specific insulin signaling, osteocalcin activation and gluco-metabolic homeostasis has proven to be complex and potentially inconsistent across animal-model systems and in humans. Moreover, the impact of postnatally acquired, osteoblast-specific insulin deficiency on the pancreas-to-skeleton-to-pancreas circuit has not been studied. To explore this relationship, we created a model of postnatal elimination of insulin signaling in osteoprogenitors. Osteoprogenitor-selective ablation of the insulin receptor was induced after ~10 weeks of age in IR^l^°^x/lox^/Osx-Cre^+/−^ genotypic male and female mice (designated postnatal-OIRKO). At ~21 weeks of age, mice were then phenotypically and metabolically characterized. Postnatal-OIRKO mice demonstrated a significant reduction in circulating concentrations of undercarboxylated osteocalcin (ucOC), in both males and females compared with control littermates. However, no differences were observed between postnatal-OIRKO and control mice in: body composition (lean or fat mass); fasting serum insulin; HbA1c; glucose dynamics during glucose tolerance testing; or in pancreatic islet area or islet morphology, demonstrating that while ucOC is impacted by insulin signaling in osteoprogenitors, there appears to be little to no relationship between osteocalcin, or its derivative (ucOC), and glucose homeostasis in this model.

## Introduction

Over the last decade, since osteocalcin was first proposed to be a regulator of glucose metabolism^1–3^, a number of controversies have arisen in the field to suggest that mouse models may not satisfactorily represent human osteocalcin physiology - and not all rodent models may fully support the overall bone-energy relationship^4–6^. Initial studies proposed that in mice, insulin signaling in osteoblasts, if disrupted *pre*natally, was linked to a feed-forward mechanism to enhance insulin secretion by activating osteocalcin to undercarboxylated osteocalcin (ucOC) through enhanced osteoclast activity, and in turn, ucOC regulated glucose homeostasis by directly enhancing pancreatic production of insulin via the GPRC6A receptor^2,3,7^. Yet, in contrast to the initial studies, when the insulin receptor (IR) is ablated prenatally at an earlier stage in ontogenesis (i.e., in osteoprogenitors), no significant abnormalities in fasting blood glucose or insulin levels, or in glucose or insulin dynamics after a glucose challenge, are observed, in either male or female mice^8^.

Therefore, to examine further this potential link between insulin signaling in osteoblastic cells and the generation of ucOC as a regulator of glucose homeostasis, we developed a mouse model designed to mitigate any potential prenatal effects that may impact this metabolic circuit through selectively eliminating the insulin receptor in osteoprogenitors only in mature mice. This model of a postnatally-acquired deficiency in insulin signaling also better mimics when insulin deficiency, insulin resistance and/or glucose dysregulation would arise in mice and humans, leading to glucose intolerance and ultimately frank diabetes. Herein, we hypothesized that disruption of insulin signaling in osteoprogenitors *in mature mice* would impact production of ucOC and potentially impact body composition and glucose homeostasis.

## Results

In mice, insulin signaling in osteoblasts has been linked by others to a feed-forward mechanism to “activate” osteocalcin into undercarboxylated osteocalcin, which, in turn, regulates glucose homeostasis by signaling through the GPRC6A on osteogenic cells^2,3,9^. To interrogate this proposed pathway in mature mice, the metabolic phenotype of mice possessing a postnatal osteoprogenitor-specific IR knock-out (postnatal-OIRKO) was characterized.

### **Weight and body composition of the postnatal-OIRKO mouse**

**Figure 1:**
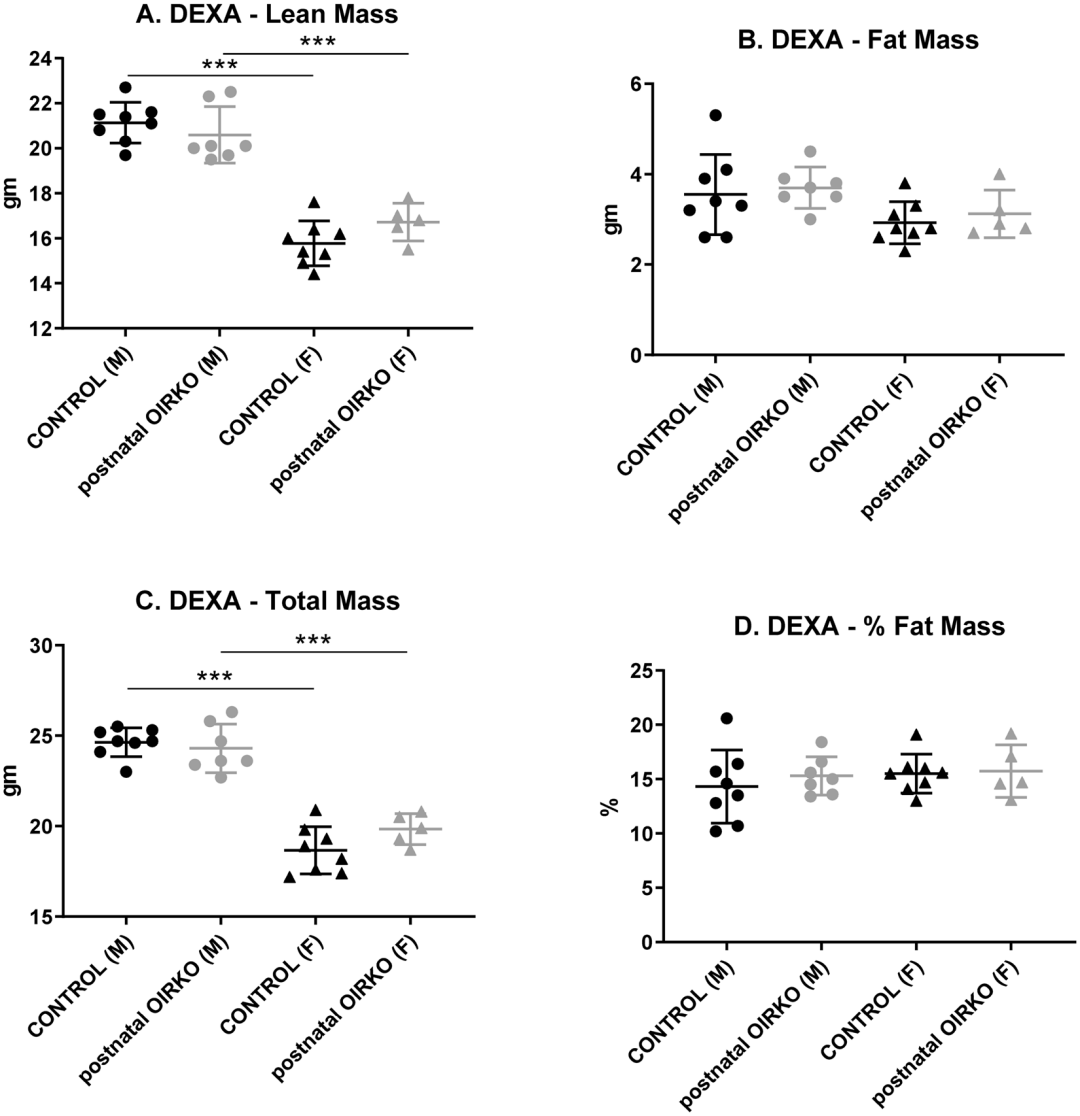
Body Composition Total body lean mass (**A**), fat mass (**B**), total mass (**C**), and percent fat mass (**D**), as analyzed by DXA, are shown for control male (M:•), OIRKO male (M:⚬), control female (F:▴), and OIRKO female (F:▵) mice. For between group comparisons, p values are designated as follows: (*), p ≤ 0.05; (**), p ≤ 0.01; (***), p ≤ 0.001.

At study end, body weight (BW) was significantly greater for males, compared with females, both for control mice and for OIRKO mice (Control: M vs. F; 28.7 ± 0.9 vs. 22.2 ± 1.4 gm, p ≤ 0.001. OIRKO: M vs. F; 28.4 ± 1.4 vs. 23.5 ± 1.0 gm, p ≤ 0.001). However, within each sex, there were no differences in BW between control and postnatal-OIRKO genotypes. Similarly, as shown in Fig. 1, body composition by DXA also demonstrated significant differences between male and female mice, both for the control and the OIRKO cohorts, for lean mass and total mass (Fig. 1A,C). Specifically, male mice consistently exhibited higher lean and total mass, compared with females. However, again, no differences were identified between control genotypes and the postnatal-OIRKO mice for either sex. Additionally, there were no significant differences between fat mass or percent (%) fat mass (Fig. 1B,D), either between males and females, or between control and OIRKO genotypes. Bone mineral density (BMD) and bone mineral content (BMC) were also assessed and no significant differences were noted between control mice and postnatal-OIRKO mice for either sex (data not shown).

### **Skeletal biomarkers in the postnatal-OIRKO mouse**

**Figure 2:**
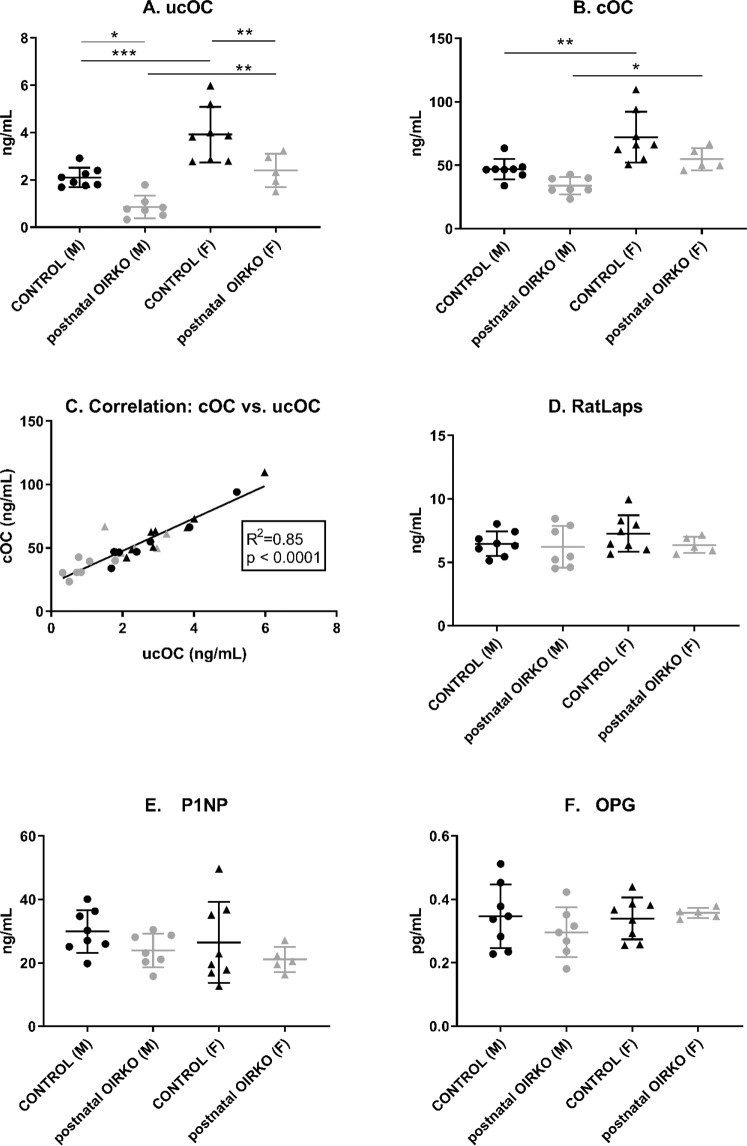
Bone Biomarkers Measurement of ucOC (**A**), cOC (**B**), RatLaps (**D**), P1NP (**E**), and OPG (**F**) are shown for control male (M:•), OIRKO male (M:⚬), control female (F:▴), and OIRKO female (F:▵) mice. For between group comparisons, p values are designated as follows: (*), p ≤ 0.05; (**), p ≤ 0.01; (***), p ≤ 0.001. A strong correlation between ucOC and cOC was present, in 2 C.

**Figure 3:**
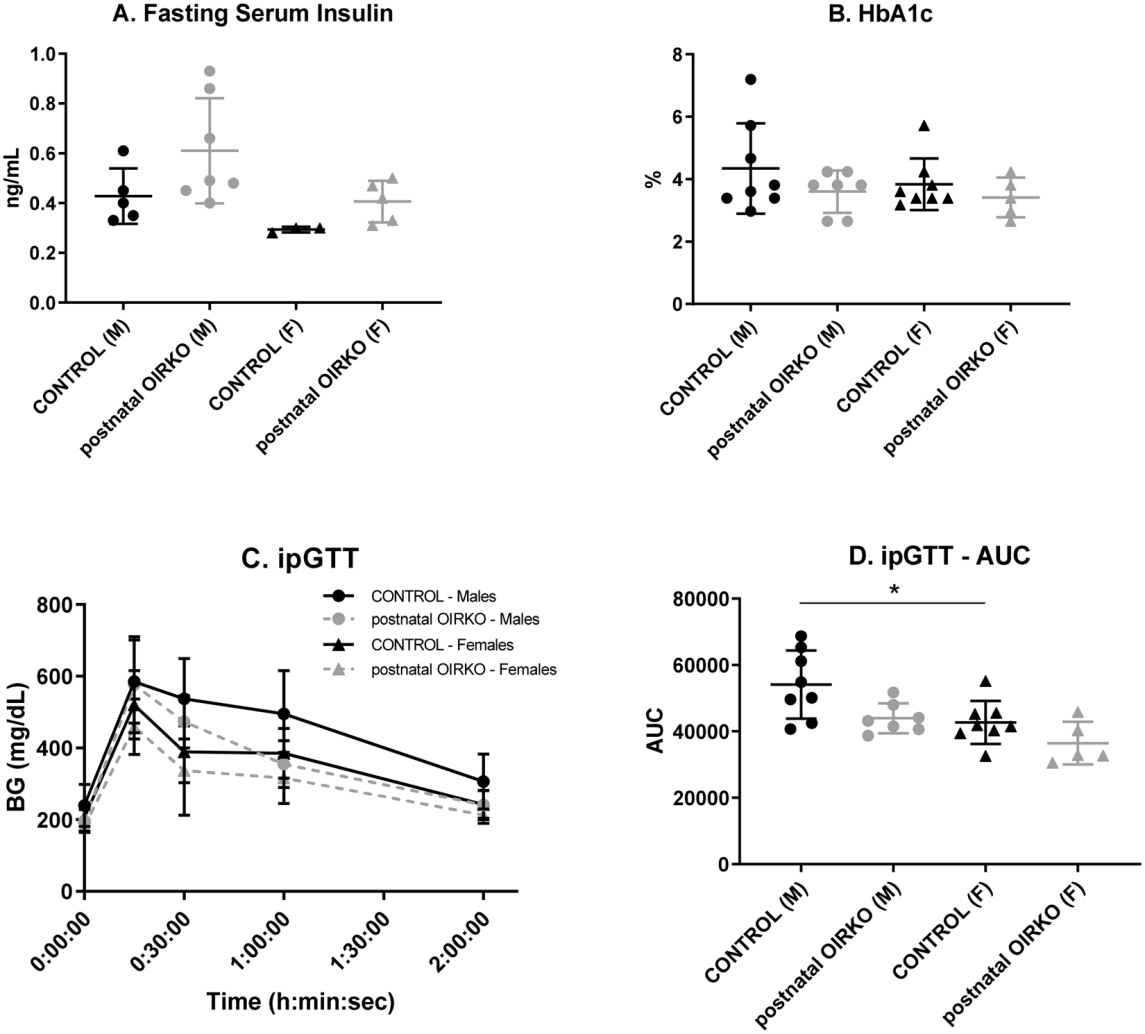
Metabolic Biomarkers Fasting serum insulin (**A**) and HbA1c (**B**) are presented, for control male (M:•), OIRKO male (M:⚬), control female (F:▴), and OIRKO female (F:▵) mice. Results for ipGTT are shown in 4 C and 4D. For between group comparisons, p values are designated as follows: (*), p ≤ 0.05; (**), p ≤ 0.01; (***), p ≤ 0.001.

As shown in Fig. 2, when comparing serum levels for both undercarboxylated (Fig. 3A, ucOC) and carboxylated osteocalcin (Fig. 2B, cOC) in males vs. females, ucOC and cOC values were significantly lower in males compared with females, whether control or OIRKO genotype (Fig. 2A,B). Moreover, for both males and females, ucOC levels were lower in OIRKO animals, compared with controls (Fig. 2A; males: p ≤ 0.05; females: p ≤ 0.01), whereas cOC did not differ between OIRKO and control mice. A strong correlation between individual ucOC and cOC values was also evident (Fig. 2C). However, serum concentrations of P1NP, a systemic biomarker of bone formation, OPG, an inhibitor of osteoclastogenesis, and RatLaps, a marker of bone resorption, did not differ across the four groups (see Fig. 2D-F).

### **Metabolic phenotyping of the postnatal-OIRKO mouse**

As shown in Fig. 3, no significant differences were identified in male or female postnatal-OIRKO mice compared to sex-matched control mice for: fasting serum insulin (Fig. 3A); fed insulin levels (data not shown); HbA1c (Fig. 3B); or glucose dynamics during glucose tolerance testing (Fig. 3C,D). Among control mice alone, ipGTT AUC was lower for females, compared with males (Fig. 3D).

### Assessment of pancreatic islets of postnatal-OIRKO mice

**Figure 4:**
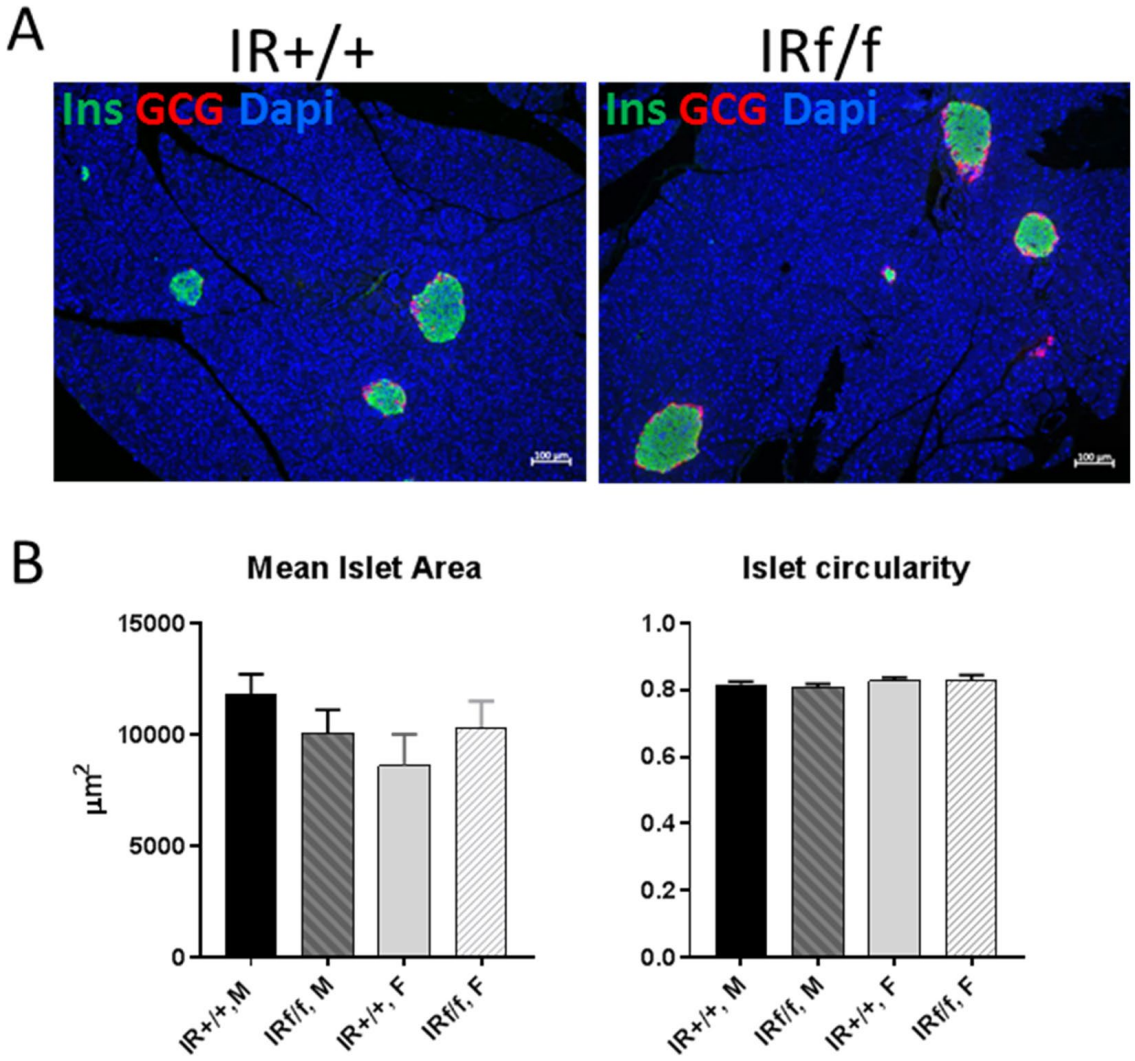
Pancreatic islet assessment. (**A**) Representative immunofluorescent microphotographs of IR^+/+^ (Wildtype) and IR^f/f^ (postnatal-OIRKO) male islets stained for Insulin (Ins, green), Glucagon (GCG, red) and Dapi (blue). Scale bar = 100 μm. (**B**) Morphometric analysis of pancreatic islets: mean islet area (left panel) and islet circularity (right panel) assessed with Zen2.3 software, in n = 60–85 islets, in n = 3 mice/group (M, male; F, female). A circularity value of 1.0 indicates a perfect circle. As the value approaches 0.0, it indicates an increasingly elongated polygon. Data are expressed as mean ± SEM.

As shown in Fig. 4A, islets from both control and postnatal-OIRKO islets stained positive for markers of β-cells (insulin) and α-cells (glucagon), suggesting no qualitative alterations in the cellular composition of islets in the postnatal-OIRKO mouse. Moreover, morphometric analysis of pancreatic islet area and islet circularity, an indirect method of estimating the overall spherical shape of islets which is correlated with islet functionality, did not demonstrate any statistically significant differences between postnatal-OIRKO and control mice (Fig. 4B,C)^10^.

## Discussion

In the postnatal-OIRKO mouse, ucOC levels were reduced in both male and female mice compared to control mice, *consistent* with previous studies^2,3^. Originally, it was proposed that OPG expression was diminished in mice in which the IR was downregulated in osteoblasts, thereby promoting osteoclastic activity which then in turn caused decarboxylation of cOC to ucOC^3^. In the current study, ucOC serum concentrations were highly correlated to cOC concentrations (Fig. 2C), but did not correlate to peripheral markers of osteoclast activity (i.e., RatLAPs) or to circulating concentrations of an inhibitor of osteoclasts, OPG (data not shown). Furthermore, RatLaps and OPG were not significantly different amongst sexes or genotypes (Fig. 2D,F). Thus the etiology of decreased ucOC in postnatal-OIRKO male and female mice is not fully understood.

The consistent finding that insulin-signaling in osteoblastic cells is involved in ucOC physiology, is also supported by findings from human studies. We and others have demonstrated that in humans with type 1 diabetes, a condition of insulin deficiency, serum concentrations of ucOC are positively associated with insulin exposure, either exogenously administered or endogenously produced, as assessed by c-peptide levels^11,12^. Because this association holds true from shortly after the diagnosis of type 1 diabetes to many years after the diagnosis, some have proposed the ucOC might be a useful surrogate marker for residual β-cell function in type 1 diabetes^12^. Post-hoc analyses examining correlations between fasting insulin levels and ucOC (R^2^ = −0.259; *p* = 0.02) or cOC (R^2^ = −0.363; *p* = 0.005) in control and postnatal OIRKO mice demonstrates an inverse correlation between fasting insulin levels and ucOC and cOC, suggesting that insulin signaling in osteoprogenitors regulates the production of ucOC and cOC.

Body composition, as determined by DXA, did not differ between control mice and postnatal-OIRKO mice. This finding differs from prior reports wherein prenatal reduction in IR expression in osteoblasts and osteoprogenitors, respectively, produced both overweight and underweight mice, respectively^2,8^. It is not clear how these variations in body weight, fat mass, and size might contribute, at least in part, to the variable metabolic phenotypes observed in each mouse model. In contrast to previous studies showing lower or normal levels of insulin in prenatal models of osteoblast or osteoprogenitor deletion of the IR, postnatal deletion showed fasting insulin levels trending higher in both male and female postnatal OIRKO mice compared to controls, consistent with other mouse models in which the IR was deleted in specific tissues, such as liver and β-cells, resulting in elevated insulin levels^13,14^. No significant differences were found in glucose dynamics in the postnatal-OIRKO mouse, similar to what was observed in the prenatal-OIRKO mouse^8^, but inconsistent with the two originally published studies which described glucose dysregulation when the IR was deleted in osteoblasts prenatally^2,3^. Furthermore, examination of pancreatic islets from control and postnatal-OIRKO mice revealed similar pancreatic islet size, architecture and morphology. This is in contrast to a prior study which revealed that when the IR was deleted prenatally in osteoblasts, islet size and morphology, as well as insulin content was diminished^3^.

Interestingly, discrepancies in glucose homeostasis have also been reported in mouse models wherein mediators of insulin signaling (i.e., FoxO transcription factors) have been eliminated in osteoblasts or osteoprogenitors. For instance, deletion of FoxO1 (which would mimic active insulin signaling) in osteoblasts, improves glucose tolerance compared to control mice and also is associated with increased insulin levels^15^. In contrast, deletion of FoxO1,3,4 in osteoprogenitors results in no alterations in insulin secretion and no dysregulation of glucose homeostasis^16^. Furthermore, variable metabolic phenotypes have been observed in models in which the purported receptor for ucOC, GPRC6A, has been disrupted. Studies in which exon II of GPRC6A was deleted demonstrated a metabolic phenotype^17^. However, later studies using different deletion constructs of GPRC6A (i.e., exon VI and full locus) have failed to demonstrate glucose dysregulation, alterations in insulin levels, or changes in body composition^18–21^.

Currently, the reasons for the variable metabolic phenotypes in the various mouse models used to explore the feed-forward mechanism of energy homeostasis and ucOC is unclear. Possible confounders may include the well-recognized variability of glucose and insulin dynamics that exists amongst inbred mouse models, between sexes, and the changes that occur over the lifespan^22,23^. Furthermore, the same genetic deletion in various inbred backgrounds have produced different metabolic phenotypes^24,25^. For instance, heterozygous knock-down of the IR and IRS1 in three different genetic backgrounds (C57BL/6, 129/Sv and DBA/2) resulted in extremely different metabolic phenotypes from frank hyperglycemia and hyperinsulinemia (C57BL/6) to essentially no metabolic phenotype (129 Sv)^25^. In addition, differences in glucose dynamics in various models and strains may only be further unmasked if the mouse model is challenged, such as may occur in diet-induced obesity^26^. Many of these potential confounders may not have been adequately controlled for in prior experimental designs. Other considerations may relate to when in development insulin signaling disruption in osteoblasts occurs (prenatally vs. postnatally); at what stage of osteoblastogenesis the disruption manifests (progenitor vs mature osteoblast, etc.); as well as possible issues with certain Cre-recombinase mouse models and the lack-of specificity for targeting only bone cells^27,28^.

## Conclusions

While there has been wide-spread acceptance of the feed-forward mechanism by which insulin signaling in osteoblasts promotes insulin production and alters glucose metabolism via ucOC through the GPRC6A receptor in β-cells, we and others have now generated several additional mouse models that bring this mechanism into question as a generalizable phenomenon. Future studies will be needed to clarify if the feed-forward mechanism is only contextual in nature and if insulin signaling throughout osteogenesis influences metabolic pathways and energy only within certain windows of osteoblastic development and on certain genetic backgrounds.

## Research Design and Methods

### Animals and experimental design

All animal procedures were approved by the Institutional Animal Care and Use Committee (IACUC) at the University of Kentucky (#2015–1348). All methods were carried out in accordance with relevant guidelines and regulations. As we have previously described^8^, osteoprogenitor-selective ablation of the insulin receptor was accomplished by crossing mice homozygous for a floxed insulin receptor (IR) allele in which loxP sites flank exon 4 of the IR gene (designated IR^lox/lox^) with heterozygous Osterix (Osx)-Cre transgenic mice (B6.Cg-Tg(Sp7-tTA,tetO-EGFP/cre)1Amc/J; The Jackson Laboratory, Bar Harbor, ME). F1 progeny were crossed to produce IR^lox/lox^/Cre^+/−^ and IR^lox/lox^/Cre^−/−^ mice which were then bred to produce experimental animals (designated Osteoprogenitor Insulin Receptor Knock-out, *OIRKO*; with genotype IR^lox/lox^/Cre^+/−^). IR^lox/lox^/Cre^−/−^ mice (*IR flox*) and IR/Cre^+/+^ mice (*Cre*+) littermates were designated as control genotypes.

Cre transgene expression is repressed by doxycycline (Tet-OFF)^29^. Therefore, for these postnatal studies, breeding animals and their progeny were maintained on chow containing doxycycline (Diet S3888, Bio-Serv; Frenchtown, NJ). After weaning, all mice were maintained on doxycycline chow until ~9–10 weeks of age; thereafter, they were transitioned to regular (no doxycycline) chow (Diet 8640, Harlan; Indianapolis, IN) for an additional 12 weeks. After switching to regular chow, the Cre transgene is expressed^30^, resulting in disruption of the floxed IR alleles (see supplementary figure 1). These mice are hereafter designated as postnatal-OIRKO.

Confirmatory genotyping was performed on all mice, using published procedures^31^. All mice were maintained in a 12-hour light-dark cycle, and provided *ad libitum* access to food and water throughout all studies, unless otherwise specified below. Individual body weights were measured weekly. Intraperitoneal (ip) glucose tolerance testing (ipGTT) was completed during the final week of the study^8,32^. For ipGTT, mice were weighed and then fasted for 4–5 hours with free access to water. Blood glucose and serum insulin levels were measured in the fasting state. Blood glucose measurements were obtained at 0, 15, 30, 60, and 120 minutes following an ip injection of glucose (1.5 g/kg body weight). Twelve weeks following removal of doxycycline from the diet, both male and female mice (mean age = 20.7 ± 0.2 weeks) from all three genotypes were then metabolically characterized. Specifically, body composition was analyzed by X-ray densitometry (DXA), as detailed below. Trunk blood was collected for physiological assessment of metabolic parameters and bone biomarkers. Pancreatic tissue was harvested at study end.

### Dual-energy X-ray absorptiometry (DXA) analysis

Body composition was measured using the Lunar PIXImus2 (GE Lunar Corp.) and software version 2.10. Instrument performance was analyzed daily by scanning an aluminum/lucite phantom (TBMD = 0.0625 g/cm2, percentage fat = 10.9%). Mice were weighed immediately prior to DXA scan. Mice were anesthetized with isoflurane and placed in a prostrate position on the imaging tray as suggested by the manufacturer. Limbs were extended from the body and were taped to the imaging tray. Heads were excluded from all analyses by placing an exclusion region of interest over the head. All scans and analyses were performed by R.C.B.

### Systemic biomarkers

Fasting or fed blood glucose (BG) was measured via glucometer (OneTouch Ultra 2 blood glucose monitoring system, Lifescan, Inc., Milpitas, CA). PINP (total procollagen type 1 N-terminal propeptide) was measured using the Rat/Mouse P1NP Competitive Enzyme Immunoassay (Immunodiagnostics Systems, Inc., Fountain Hills, AZ; #AC-33F1). RatLaps (C-terminal telopeptide I) and OPG (osteoprotegerin) were measured by rodent-specific ELISA (Immunodiagnostics Systems, Inc., Fountain Hills, AZ, AC-06F1; and RayBiotech Inc., Norcross, GA, ELM-OPG-1, respectively). Similarly, mouse osteocalcin components [carboxylated osteocalcin (cOC) and incompletely carboxylated or undercarboxylated osteocalcin (ucOC)] were measured by ELISA (MyBioSource.com, San Diego, CA; #MBS744268 and #MBS706251, respectively). Fasting insulin (measured at baseline during ipGTT), was quantified using a mouse ultrasensitive insulin ELISA (Crystal Chem USA, Elk Grove Village, IL; #90080).

### Pancreatic islet histology

#### Islet morphometry

Islet area (μm^2^) and circularity were assessed by using Hematoxylin & Eosin stained sections of the pancreas (5 um thickness), from n = 3 mice from each group. Three sections per mouse (100 μm apart) were photographed with the AxioObserver Z1 inverted microscope (10x magnification) and analyzed with the ZEN 2.3 software (Carl Zeiss Microscopy, Jena, Germany).

#### Immunofluorescence

Pancreatic tissue was fixed overnight in 4% phosphate-buffered paraformaldehyde, paraffin-embedded and sectioned on a classical microtome (5 μm thickness). The sections were collected on Superfrost Plus coverslips, dewaxed in xylene, and rehydrated through a series of ethanol baths. After antigen retrieval (citrate buffer 10 mM, pH = 6), the sections were blocked with normal goat serum (Vector Laboratories, Burlingame, CA) and incubated overnight at 4 ^o^C with the primary antibodies: mouse anti-Insulin (Sigma-Aldrich Corp., St. Louis, MO; 1:600) and rabbit anti-Glucagon (Abcam, Cambridge, MA; 1:400). The sections were than incubated at room temperature with a cocktail of secondary antibodies conjugated with Alexa594 and Alexa488 (Invitrogen Alexa Fluor dye-labeled secondary antibodies, Thermo Fisher Scientific, Waltham, MA; 1:200). Nuclei were stained with DAPI (Sigma-Aldrich Corp., St. Louis, MO). Negative controls were obtained by omitting the primary antibody. The sections were examined with the AxioObserver Z1 inverted microscope (Carl Zeiss Microscopy, Jena, Germany) equipped with an Axiocam 506 monochrome camera. Images were captured, recorded, and analyzed using the ZEN 2.3 Pro or ImageJ software.

### Statistical analysis

Unless otherwise stated, results for individual parameters are reported as mean ± standard deviation (SD). A one-way Analysis of Variance (ANOVA) was used to determine differences between groups for each response variable, followed by the Tukey test to correct for multiple comparisons; statistically significant multiplicity-adjusted *p* values for each comparison were reported, and values were considered statistically significant at *p* ≤ 0.05. Where applicable, to determine the relationship between variables of interest, a Pearson correlation coefficient was computed between data sets, and the two-tailed *p*-value and R^2^ are reported.

## Supplementary information

Supplementary information.
