# Supplementary material for: Postnatal loss of the insulin receptor in osteoprogenitor cells does not impart a metabolic phenotype
Source: Sci Rep. 2020 Jun 1;10:8842. doi: 10.1038/s41598-020-65717-3 (PMC7264347; doi:10.1038/s41598-020-65717-3)
Supplement: Supplementary file 1 — Supplementary information. [file 41598_2020_65717_MOESM1_ESM.docx]

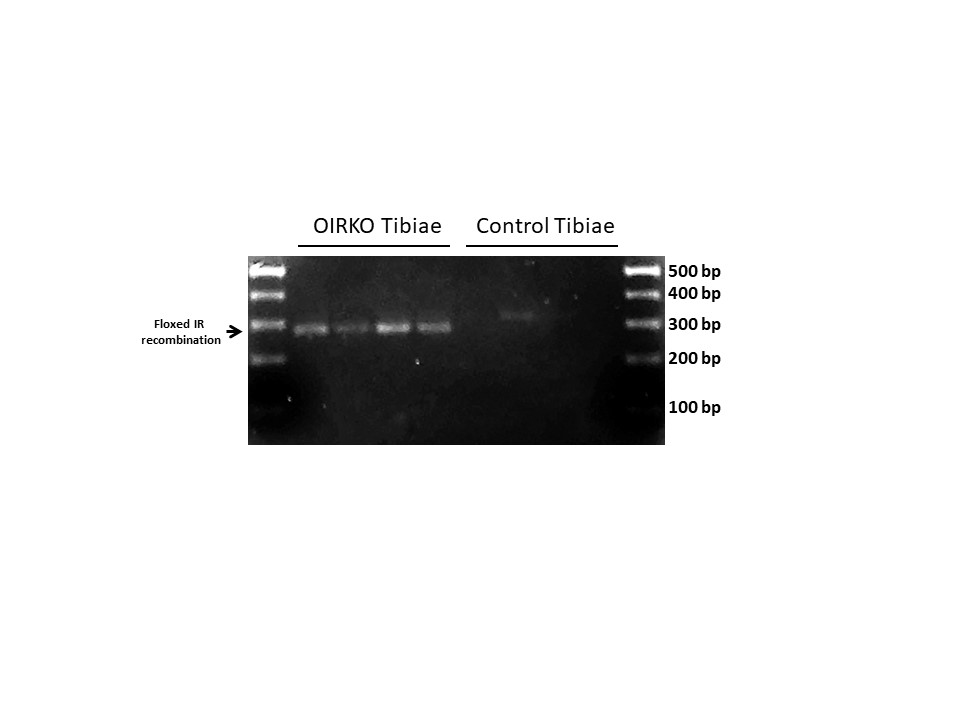


*Supplemental Figure 1 -* Postnatal-ablation of the IR in osteoprogenitors was achieved by taking mature (>10 weeks old) IR^lox/lox^/Cre^+/-^ mice off doxycycline for 12 weeks to induce Cre and thereby excise the targeted IR receptor. PCR data from tibiae of postnatal control and postnatal-OIRKO mice *off* doxycycline for 12 weeks, demonstrating that the IR allele is deleted only in the *IR^lox/lox^ /Cre*^+/-^ genotype (arrow).
